# Replacement of Fish Meal by Black Soldier Fly (*Hermetia illucens*) Larvae Meal: Effects on Growth, Haematology, and Skin Mucus Immunity of Nile Tilapia, *Oreochromis niloticus*

**DOI:** 10.3390/ani11010193

**Published:** 2021-01-15

**Authors:** Nisarat Tippayadara, Mahmoud A. O. Dawood, Patcharin Krutmuang, Seyed Hosseini Hoseinifar, Hien Van Doan, Marina Paolucci

**Affiliations:** 1 Faculty of interdisciplinary Studies, Khon Kaen University, Nong Khai 43000, Thailand; nisati@kku.ac.th; 2 Department of Animal Production, Faculty of Agriculture, Kafrelsheikh University, Kafrelsheikh 33516, Egypt; mahmouddawood55@gmail.com; 3 Department of Entomology and Plant Pathology, Faculty of Agriculture, Chiang Mai University, Chiang Mai 50200, Thailand; patcharin.k@cmu.ac.th; 4 Department of Fisheries Gorgan, University of Agricultural Sciences and Natural Resources, Gorgan, Iran; hossein.hoseinifar@gmail.com; 5 Department of Animal and Aquatic Sciences, Faculty of Agriculture, Chiang Mai University, Chiang Mai 50200, Thailand; 6 Department of Sciences and Technologies, University of Sannio, 82100, Benevento, Italy; paolucci@unisannio.it

**Keywords:** sustainable aquaculture, fish meal, black soldier fly larvae meal, *Hermetia illucens*, Nile tilapia, *Oreochromis niloticus*, growth performance, haematological parameters, skin mucus immunity

## Abstract

**Simple Summary:**

Fish meal (FM) is the primary ingredient of the farmed fish’s diet. However, the decline in wild fish catches, and the growing demand for aquaculture feed have resulted in a dramatic reduction of FM supply. Thus, it is essential to seek for alternatives, such as insect meal (IM), to support sustainable aquafeed production. Among insects, the black soldier fly larvae are promising because they are rich in essential amino acids, minerals, and vitamins. Therefore, the present study was performed to assess the effects of IM as a partial or total replacement of FM on the growth and hematological parameters and skin mucus immunity of Nile tilapia. Growth and feed utilization efficiency indices, feed intake, survival rates, and hematological parameters were not significantly different between FM and IM fed fish, while the mucosal immune response was improved in IM fed fish. In conclusion, these results show that IM can be used to substitute FM in the Nile tilapia diet. These findings can be used to develop alternative aquafeed for sustainable aquaculture.

**Abstract:**

Fish meal (FM) is no longer a sustainable source for the increasing aquaculture industry. Animal proteins from insects may be used as a FM alternative source as long as they do not create adverse effects in fish. Black soldier fly larvae meal (BSFLM) was tested in a 12-week experiment on Nile tilapia (*Oreochromis niloticus*). Four hundred and twenty (14.77 ± 2.09 g) fish were divided into seven groups and were fed seven diets: control (0% BSFLM-100% FM), and FM replaced by BSFLM at rates of 10%, 20%, 40%, 60%, 80% and 100%. Growth indexes, feed utilization efficiency indices, feed intake, and survival rate were not significantly different (*p* > 0.05) between FM and BSFLM fed fish. Values of red blood cell, white blood cells, hemoglobin, hematocrit, mean corpuscular volume and hemoglobin, mean corpuscular hemoglobin concentration, red blood cell distribution width, and platelet values were not affected by BSFLM. Skin, mucus lysozyme, and peroxidase activities were improved in BSFLM fed fish. BSFLM can be used as a substitution for FM in the Nile tilapia (*O. niloticus*) diet at up to a 100% rate with no adverse effects.

## 1. Introduction

The aquaculture sector increases by around 5.8% yearly because of rapid expansion and intensification across the industry [1]. Fishmeal (FM) has been used as the primary protein ingredient in aquaculture for decades because of its balanced essential amino acids, easy digestibility, and palatability, crucial attributes to enhance nutrient digestion and absorption [2]. Nevertheless, the gradual decline in wild fish catches, and the growing demand for aquaculture feed resulted in a dramatically reduced supply of FM and the elevation of diet prices [3,4]. FM is believed to no longer be capable of supporting the development of the aquaculture industry in the coming years [5,6]. This raises the need to seek better alternatives to FM for sustainable aquafeed production [7].

Protein sources of animal origins, such as insect meal (IM), can be used as alternative sources for fish meal in aqua-feed [8,9,10]. In recent years, interest in the study of IM in fish farming as a feasible alternative to feed has risen dramatically [11,12]. IM is a good source of protein, minerals, and vitamins, similar to FM [13]. It is also rich in essential amino acids, especially lysine, methionine, and leucine, containing no anti-nutritional elements [14,15]. Among insects, the black soldier fly larvae (BSFL) are particularly promising because of their ability to turn food waste into premium protein, and as such mass production has increased over recent years [7,8]. The BSFL contain about 30–58% protein and 10–30% lipids and essential amino acids, similar to FM [16,17,18]. They also contain many macro- and micro- minerals, as well as valuable vitamins [19].

The partial or total replacement of dietary FM with BSFL has been successfully demonstrated in various fish species, such as: rainbow trout, *Oncorhynchus mykiss* [20]; Japanese seabass, *Lateolabrax japonicus* [21]; Atlantic salmon, *Salmo salar* [8,10]; European sea bass, *Dicentrarchus labrax* [7]; hybrid tilapia (Nile × Mozambique, *Oreochromis niloticus* × *O. mozambique*) [22]; marron, *Cherax cainii* [23]; and rice field eel, *Monopterus albus* [24]. However, as far as we know, there is limited information regarding the effects of black soldier fly larvae meal (BSFLM) on the growth, hematology, and skin mucus of Nile tilapia, which occupies the second highest position in the world in terms of production due to its high demand, rapid growth, and fair prices [25,26]. Therefore, the present study was performed to assess the effects of using BSFLM as a partial or total replacement for dietary FM on the growth, hematology, and skin mucus immunity of Nile tilapia, *O. niloticus*.

## 2. Materials and Methods

### 2.1. Black Soldier Fly Larvae Meal (BSFLM) Preparation

Black soldier fly larvae (*Hermetia illucens*) were provided by Prof. Dr. Patcharin Krutmuang, Department of Entomology and Plant Pathology, Faculty of Agriculture, Chiang Mai University, Chiang Mai 50200, Thailand. They were dried in a hot air oven at 50 °C for 24 h. They were then ground into a fine powder and kept at 4 °C for further use.

### 2.2. Experimental Diets

The basal diet, which has been demonstrated to be suitable for Nile tilapia [27] was prepared with the substitution of FM with BSFLM: 0 (Diet 1—control), 10% (Diet 2), 20% (Diet 3), Diet 4 (40%), Diet 5 (60%), Diet 6 (80), and Diet 7 (100%). Ingredients and proximate composition of the basal diet and proximate composition of BSFLM are given in Table 1 and Table 2, respectively. Powdered feed obtained from Baan Pramong Company Limited, Bangrabow, Ban Sang, Prachinburi, 25150, Thailand was completely mixed into the manufacturing of feed pellets, and soybean oil and water were added to make a stiff dough. It was then moved to form the pellets through an extruder at a temperature of 100 °C. The wet pellets (size 2 mm) were then collected and dehydrated in a 50 °C hot air oven to achieve a moisture content of three percent, then placed in plastic bags and stored at 4 °C. The composition of the diets was analyzed following the method of AOAC-Association of Official Analytical Chemists [28].

### 2.3. Experimental Procedure

Chitralada 3 Nile tilapia (*O. niloticus*) fingerlings were purchased from Inland Aquaculture Research and Development Division, Department of Fisheries, Thailand. Fish were fed a commercial feed from Charoen Pokphand Foods Public Company Limited (CP, 9950) for 4 weeks and a basal diet for 15 days. Afterward, 420 fish (14.77 ± 2.09 g fish^−1^) were later captured and distributed into 21 glass tanks (volume 100 L tank^−1^) at a density of 20 fish/tank. Each aquarium was supplied with continuous aeration via compressed air. Fish were divided into 7 treatments in triplicates and fed on tested diets up to apparent satiation at 9:00 and 16:00 h for 12 weeks. The light was maintained at a 12:12 h light:dark cycle with natural light. To maintain clear and healthy water throughout the experimental period, three-quarters of the aquarium’s water was siphoned daily to remove feces and uneaten food and was replaced with clean well-aerated water from a storage tank.

### 2.4. Water Quality Measurement

Water quality assessment was conducted every two weeks. Water temperature and dissolved oxygen were measured using a YSI Model 52 meter. pH and NH4+ + NH3 were measured using an IQ scientific meter and Phenate-hypochlorite following the method of [29]. The TSD and conductivity were measured using HI 98311 (Hanna Instruments, Bangkok, Thailand). The temperature (0 °C), conductivity (µS/cm), TDS (mg/L), dissolved oxygen (mg/L), pH, and total ammonia (mg/L) were 28.93 ± 1.60, 341.93 ± 31.07, 135.25 ± 21.76, 5.41 ± 0.23, 7.30 ± 0.17, and 0.09 ± 0.07, respectively.

### 2.5. Sample Collections

#### 2.5.1. Blood Collection and Hematological Parameters

Fish were fasted for 24 h prior to the blood collection and anesthetized using clove oil (5 mL L^−1^). Then, one mL of blood was taken from the fish’s caudal vein (15 fish per treatment). The anticoagulant was heparin sodium. The blood with the anticoagulant was immediately transferred into a 1.8 Eppendorf tube and stored at 4 °C for further analysis. The red blood cell (RBC), hemoglobin (Hb), hematocrit (HCT), mean corpuscular volume (MCV), mean corpuscular hemoglobin (MCH), mean corpuscular hemoglobin concentration (MCHC), red blood cell distribution width (RDW-CV), and platelet (PLT) values were measured via a blood cell analyzer (Sysmex/XN-1000 S/N 19393, Meditop, Soi Lat Phrao, Thailand). The measurement of RBC and white blood cell (WBC) counts were performed as described in [30], while differential counts of lymphocytes, monocytes, and neutrophils were detected by smears stained with Wright Giemsa.

#### 2.5.2. Skin Mucus Preparation

Skin mucus was collected from 3 fish and pooled as reported in [31]. Briefly, the anesthetized fish with clove oil (5 mL L^−1^ of water) were put in a polyethene bag containing 10 mL of 50 mM NaCl. Fish were gently rubbed inside the bag for two minutes. Afterward, the solution was immediately released into a 15 mL sterile tube and centrifuged at 1500× *g* at 4 °C for ten minutes (5810R Eppendorf, Engelsdorf, Germany). Then, 500 µL of supernatant were gathered and kept at −80 °C for further analysis. 

### 2.6. Growth Parameter Calculations

At every 2 weeks interval, all fish were fasted for 24 h, and then growth parameters and survival rates were determined using the following formulae: Daily weight gain (DWG) = (mean final weigh − tcmean initial weight) ÷ t (days); Weight gain (WG) = (mean final weight − mean initial weight); Specific growth rate (SGR %/day) = 100 × (lnWt − lnWo) ÷ t (days); Relative growth rate (RGR%) = [Wf (final weight) − Wi (initial weight)]/Wf × 100; Food conversion ratio (FCR) = Total amount of the feed consumed (g)/Wet weight gain; Feed efficiency (FE%) = (1/FCR) × 100, and Survival: (SR%) = 100 (Nf ÷ Ni) with Nf and Ni: final and initial number of fish.

### 2.7. Fish Morphometric Indices

After exsanguination, 15 fish per treatment were dissected for hepatopancreas and viscera collection. After that, they were placed in saline solution (0.86%) and stored at −20 °C. The fish’s morphometric indices were calculated as the following equations: Condition factor (CF) = 100 × BW in g/(TL in cm^3^); Hepatosomatic index (HSI) = 100 × (liver weight (g)/whole fish weight (g)); Viscerosomatic index (VSI) = 100 × (viscera weight (g)/whole fish weight (g)).

### 2.8. Digestible Efficiency Measurement

Apparent digestibility coefficients were determined following the method reported in [32] with the use of 0.5% chromic oxide as a marker. Fish’s feces in each tank were collected and stored at −20 °C, and then oven-dried at 50 °C for 48 h. The dried feces were used for analyzing chromic oxide and nutrients, according to the method described in [33]. Apparent digestibility coefficients (ADC) were measured by the following equation ADCdiet = [1 − (dietary Cr_2_O_3_ level × feces nutrient or energy level: feces Cr_2_O_3_ level × dietary nutrient or energy level)] × 100.

### 2.9. Mucosal Immune Responses

#### 2.9.1. Skin Mucus Lysozyme Assay

Skin mucus lysozyme was determined using the method reported in [34] with slight modifications, as mentioned in [35]. Briefly, 100 µL of skin mucus from each fish were loaded into 96 well-plates, in triplicate. *Micrococcus lysodeikticus* (100 µL, 0.3 mg mL^−1^ in 0.1 M citrate phosphate buffer, pH 5.8; Sigma-Aldrich, Co Ltd, Bangkok, Thailand ) solution was loaded into each well and gently mixed. The change in turbidity was recorded every 30 s for 10 min at 540 nm, 25 °C using a microplate reader. The sample’s equivalent unit of activity was determined and compared with the standard, and expressed as µg mL^−1^ serum.

#### 2.9.2. Skin Mucus Peroxidase Assay

Peroxidase activity was performed using the protocol reported in [36] with modification as mentioned in [35]. Briefly, 5 µL of skin mucus from each fish were loaded into 96 flat-bottomed well-plates in triplicate. Then, 45 µL of Hank’s Balanced Salt Solution (without Ca^+2^ or Mg^+2^) and 100 µL of solution (contains 40 mL of distilled water + 10 µL of H_2_O_2_, 30%; Sigma Aldrich + one pill of 3,3’,5,5’-tetramethylbenzidine, TMB; Sigma Aldrich) were added into each well. Once the reaction color turned blue (30–60 s), 50 µL of 2 M H_2_SO_4_ were added to each well. The optical density was read at 450 nm by a microplate reader (Synergy H1, BioTek, Winooski, VT, USA, USA). Samples not containing serum or skin mucus were considered to be blanks. A single unit was defined as the amount which produced an absorbance change, expressed as units (U) mL^−1^ of serum or mucus.

### 2.10. Statistical Analysis

One-way variance analysis (ANOVA) and Duncan’s Multiple Range Test) using SAS software, 2003 were applied for data analysis after checking the normality of the data by the Kolmogorov-Smirnov test. Various mean values (*p* < 0.05) and other measurements are shown as mean ± SD. The optimum BSFLM level was determined using quadratic and linear regression analyses [37].

## 3. Results

### 3.1. Growth Performance

Growth and feed utilization parameters are displayed in Table 3 and Table 4. The results indicate that the highest growth parameters were observed in fish Diets 3 and 4 (Table 3). 

However, no significant differences were recorded between black soldier fly larvae meal (BSFLM) and the control diets. No differences in appetite were detected between fish fed on modified diets and controls. Moreover, no significant differences in total feed intake, and rate of fish intake, fish conversion ratio, feed efficiency, and total digestibility were displayed between the control and BSFLM substitution fed fish (Table 4). In con trast, a significant increase in apparent protein digestibility coefficient was observed in fish fed BSFLM compared to the control, and the highest value was observed in fish fed Diet 7 (Table 4).

The fish’s morphometric indices show that no noticeable discrepancies in condition factor (CF), hepatosomatic (HSI), and viscerosomatic indexes (VSI) between the control and BSFLM substitution diets (Table 5). However, the final total length was significantly improved in fish fed Diet 3 (Table 5). 

**Figure 1:**
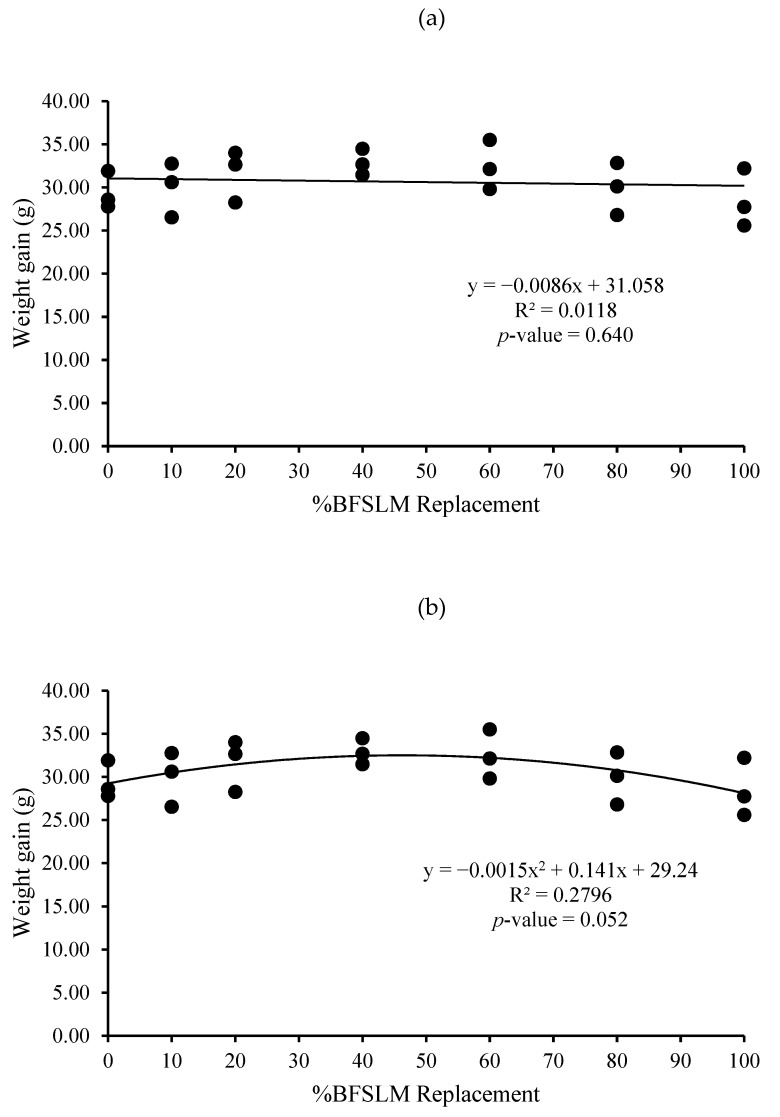
Linear (**a**) and quadratic (**b**) estimate: When X = % Black soldier fly larvae meal (BSFLM) replacement; Y = weight gained after 12 weeks of feeding with control (0% BSFLM and 100% fish meal (FM)), and FM replaced at 10%, 20%, 40%, 60%, 80%, and 100% of the BSFLM.

**Figure 2:**
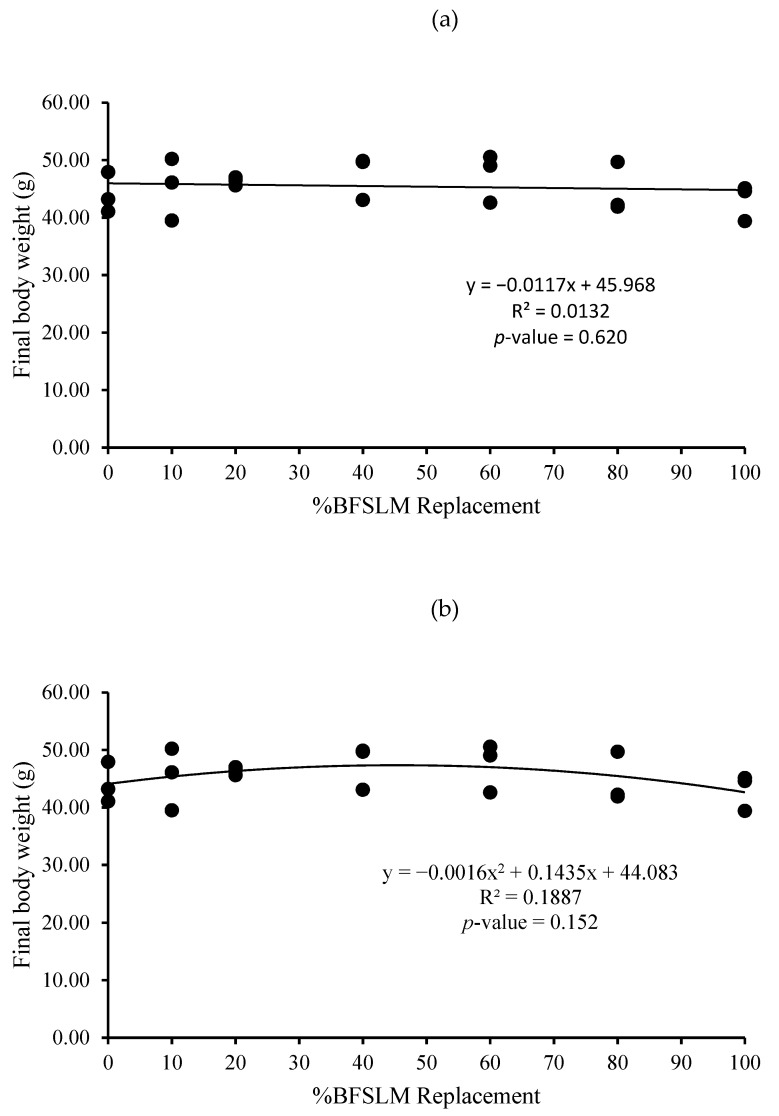
Linear (**a**) and quadratic (**b**) estimate: When X = %BSFLM replacement; Y = Final body weight after 12 weeks of feeding with control (0% BSFLM and 100% FM), and FM replaced at 10%, 20%, 40%, 60%, 80%, and 100% of the BSFLM.

**Figure 3:**
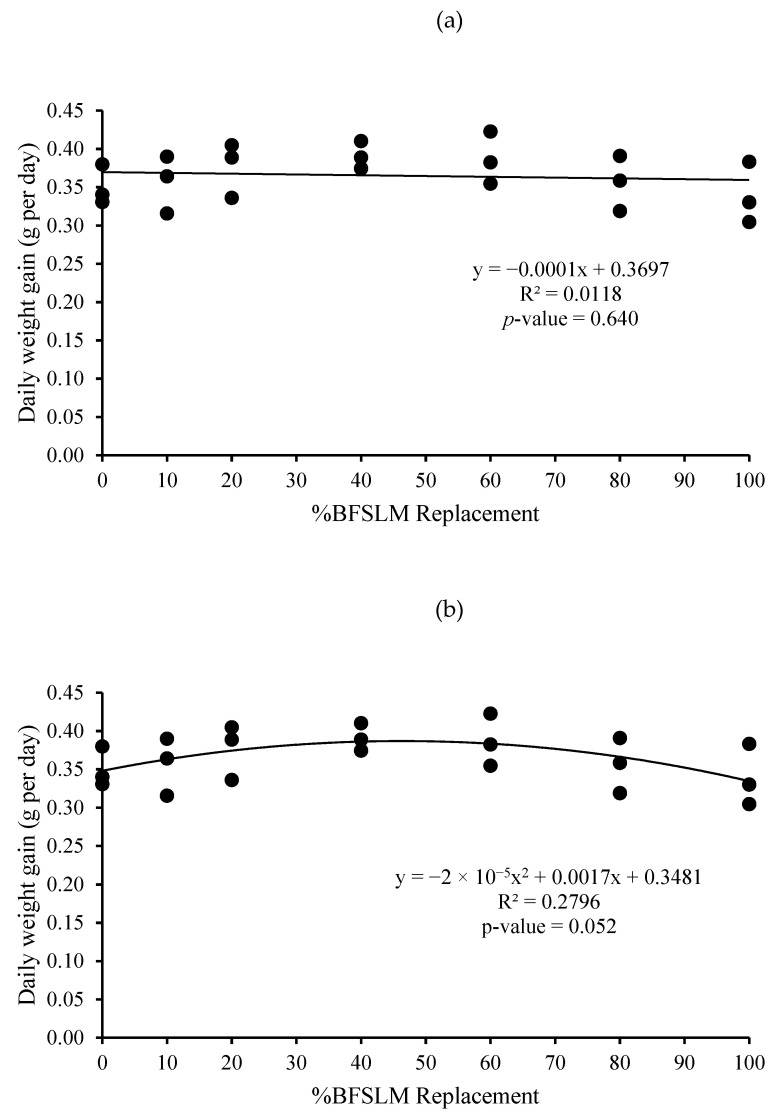
Linear (**a**) and quadratic (**b**) estimate: When X = %BSFLM replacement; Y = Daily weight gain after 12 weeks of feeding with control (0% BSFLM and 100% FM), and FM replaced at 10%, 20%, 40%, 60%, 80%, and 100% of the BSFLM.

**Figure 4:**
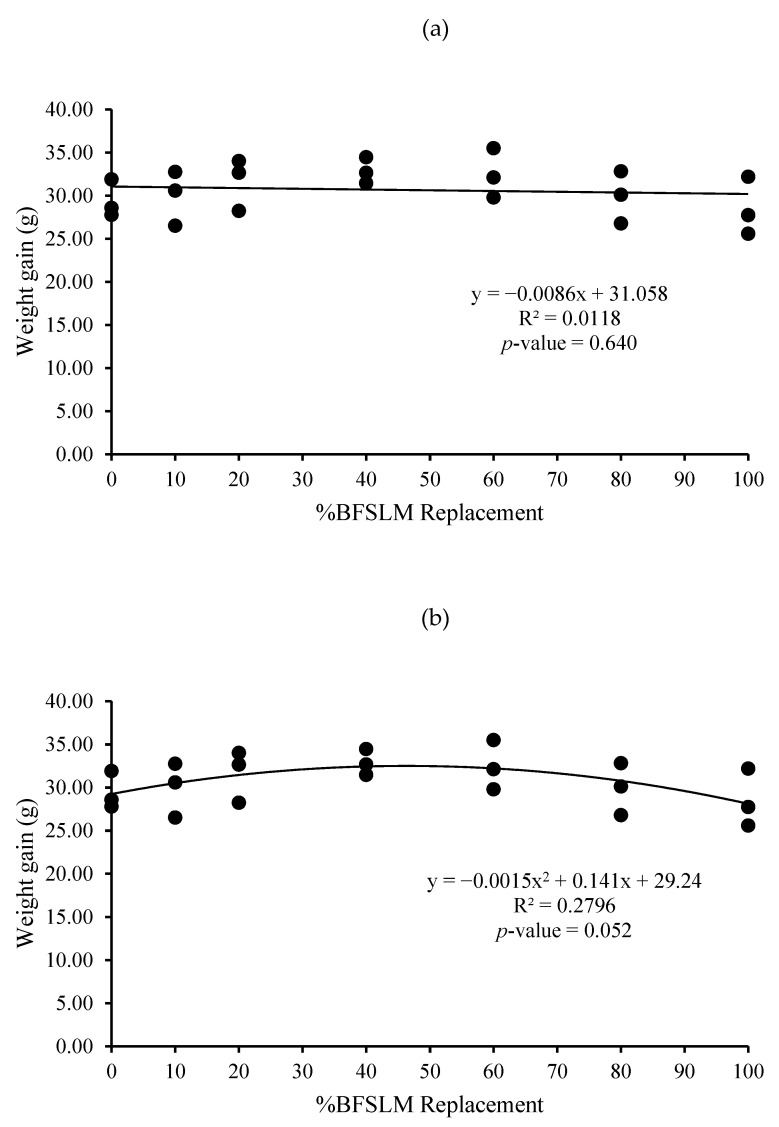
Linear (**a**) and quadratic (**b**) estimate: When X = %BSFLM replacement; Y = Weight gain after 12 weeks of feeding with control (0% BSFLM and 100% FM), and FM replaced at 10%, 20%, 40%, 60%, 80%, and 100% of the BSFLM.

**Figure 5:**
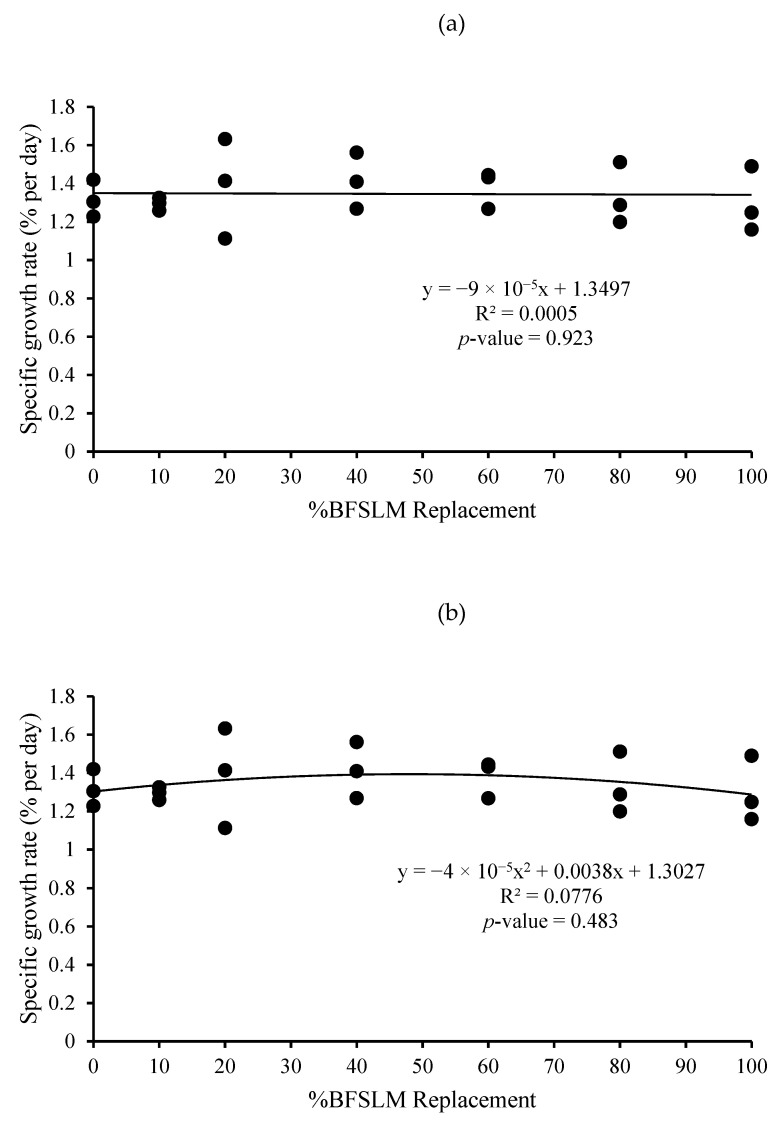
Linear (**a**) and quadratic (**b**) estimate: When X = %BSFLM replacement; Y = Specific growth rate after 12 weeks of feeding with control (0% BSFLM and 100% FM), and FM replaced at 10%, 20%, 40%, 60%, 80%, and 100% of the BSFLM.

**Figure 6:**
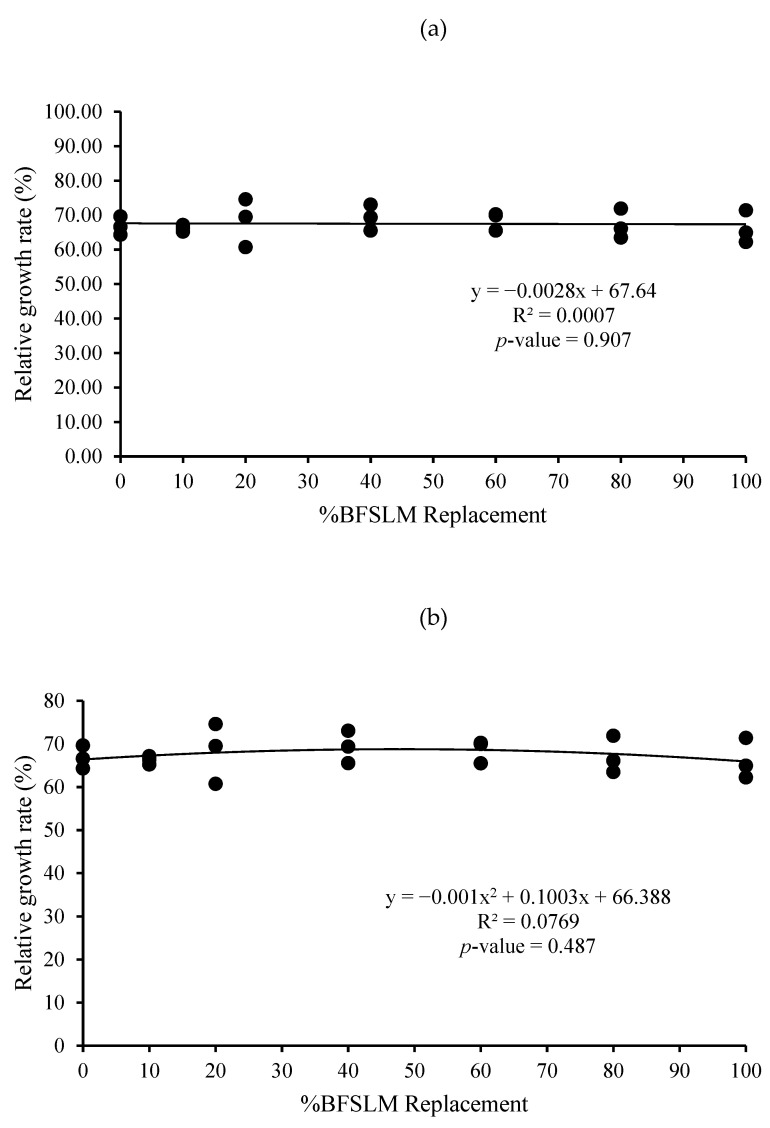
Linear (**a**) and quadratic (**b**) estimate: When X = %BSFLM replacement; Y = Relative growth rate after 12 weeks of feeding with control (0% BSFLM and 100% FM), and FM replaced at 10%, 20%, 40%, 60%, 80%, and 100% of the BSFLM.

The optimum BSFLM was 47% based on quadratic regression of weight gain, final body weight, daily weight gain, specific growth rate, and relative growth rate. However, the linear regression showed no significant differences (Figure 1, Figure 2, Figure 3, Figure 4, Figure 5 and Figure 6).

### 3.2. Blood Parameters

The blood parameters of Nile tilapia fed BSFLM are illustrated in Table 6 and Table 7. The results reveal that there were no significant differences in Red blood cell (RBC), hemoglobin (Hb), hematocrit (HCT), mean corpuscular volume (MCV), mean corpuscular hemoglobin (MCH), mean corpuscular hemoglobin concentration (MCHC), red blood cell distribution width (RDW-CV), and platelet (PLT). values between the control and BSFLM fed fish (Table 6).

### 3.3. Skin Mucus Immune Response

Skin mucus lysozyme (SMLA) and skin mucus peroxidase (SMPA) activities of fish fed BSFLM substitution diets are illustrated in Table 8. The results show that BSFLM diets significantly (*p* ≤ 0.05) stimulated SMLA and SMPA after 12 weeks of feeding with the maximum amounts recorded in Diet 4 and 5 compared to the control. No significant differences in SMLA and SMPA (*p* ≥ 0.05) were detected in fish fed diets 2, 3, 6, and 7.

## 4. Discussion

Fish nutritionists focus on the feeding strategies required for the optimal growth of Nile tilapia in particular due to its popularity as an affordable, nutritious, and cheap source of animal proteins [38]. The use of black soldier fly larvae meal (BSFLM) as a potential source of protein in tilapia diets has been abundantly investigated [7,22,24,39]. Concurrently, the results of the present study illustrate that fish fed up to 100% of the BSFLM inclusion level (total replacement of fish meal (FM)) had no adverse effects on the growth performance, somatic indices, and survival rate of Nile tilapia. In this context, Rana, Salam, Hashem, and Islam [40] elucidated that mono-sex tilapia fed diets with BSFLM replaced with 50% of FM displayed similar growth performance to the control group. Similarly, Ushakova, Ponomarev, Bakaneva, Fedorovykh, Levina, Kotel’nikov, Kotel’nikova, Bastrakov, Kozlova, and Pavlov [41] reported that feeding Mozambique tilapia with dried flour of BSFLM pre-pupae in a dose of 0.5 g kg^−1^ of feed for 30 days resulted in a significant increase of average daily gain with no significant differences in the survival rate. Additionally, Dietz and Liebert [42] reported that the inclusion of 50% BSFLM as a replacer for soy protein-concentrate did not compromise the growth performance and feed conversion ratio (FCR) of Nile tilapia. Interestingly, when FM was replaced with 50% of BSFLM, the results displayed no adverse effects on the growth performance of Nile tilapia [43]. The replacement of 50% of the FM with a mixture of BSFLM and *Manihot esculenta* leaf meal resulted in increased growth of Nile tilapia [44]. Likewise, Devic, Leschen, Murray, and Little [45] observed no adverse effects on the growth performance of Nile tilapia fed up to 80 g BSF/kg diet. Toriz–Roldan, Ruiz–Vega, García–Ulloa, Hernández–Llamas, Fonseca–Madrigal, and Rodríguez–González [46] illustrated that dietary inclusion of BSFLM at the rate of 6% did not affect the growth performance; however, the protein efficiency ratio was enhanced. More recently, Fisher, Collins, Hanson, Mason, Colombo, and Anderson [8] indicated that Atlantic salmon fed diets containing up 200 g kg^−1^ of BSFLM showed growth performances similar to the control. Similarly, no significant difference in the fish growth and survival rate was recorded in European sea bass (*Dicentrarchus labrax*) that was fed BSF at rates of up to 50% [7]. Li, Kortner, Chikwati, Belghit, Lock, and Krogdahl [10] reported that total substitution of FM with BSFLM does not compromise the gut health of seawater phase Atlantic salmon.

The measured feed efficiency indices, such as total feed intake, rates of feed intake, feed conversion ratio, feed efficiency, and total digestibility were similar among tilapia groups that were fed different levels of BSFLM. These results were similar to previous results reported in rainbow trout (*Oncorhynchus mykiss*) [20,47], Atlantic salmon (*S. salar*) [8,10,48], Japanese seabass (*Lateolabrax japonicas*) [21], zebrafish (*Danio rerio*) [49], and European sea bass (*D. labrax*) [7,50]. Interestingly, a significantly higher apparent protein digestibility coefficient was observed in fish fed BSFLM compared to the control diets, and the highest value was observed in fish fed Diet 7. The results were in disagreement with a previous study, which showed that the apparent digestibility coefficient of crude protein was lowest in fish fed insect meal diets [51]. The presence of chitin in insect meal might interfere with the utilization of protein [52,53]. Nonetheless, several investigations demonstrate that chitinolytic enzyme activities were found in the organs of some fish, such as gastric mucosa, intestinal mucosa, pyloric caeca, and pancreas [54,55,56,57]. Nile tilapia, an omnivorous species with a great ability to fed on plankton, may possess some advantages in chitin degradation and digestion [53,58,59]. The feeding nature and significant intake of chitin make it likely that chitinolytic enzymes play an important role for tilapia digestive physiology [53]. Moreover, the dietary inclusion of chitin could increase gut microflora diversity and act against several harmful bacteria, such as *Escherichia coli, Anaerorhabdus furcosa,* and *Aeromonas hydropila* [60,61,62,63,64]. Additionally, it has been reported that BSFLM is a rich source of omega 3,6, and 9 [15,65,66,67,68,69], a composition that may improve the growth performance of the host. Based on quadratic regression analysis, the optimal BSFLM level was 47%. However, it is important to underline that the lowest *p*-value for the quadratic analysis is *p* = 0.052, slightly above the *p* < 0.05 limit considered "significant". The optimal BSFLM level is higher than in Atlantic salmon (*Salmo salar*) (12.5%) [39], hybrid tilapia (Nile × Mozambique, *Oreocromis niloticus × O. mozambique*) (30%) [22], and rice field eel (*Monopterus albus*) (15.78%) [24]; however, it is lower than in European sea bass (*D. labrax*) and grass carp (*Ctenopharyngodon idellus*) (50%) [7,70] and Japanese seabass (*L. japonicus*) (64%) [21].

Hematological indices of fish are regarded as essential measurements for evaluating the general health status and physiological stress responses of fish fed formulated rations [71]. Herein, the impacts of feeding BSFLM on several hematological indices of tilapia were evaluated. The results displayed that the inclusion of BSFLM did not influence the redblood cells (RBCs) or the white blood cells (WBCs). Also, Zhou, Liu, Ji, and Yu [72] found that replacement of FM by BSFLM at 35, 70, 105, and 140 g BSFLM/kg had no effects on Jian carp’s glucose, total protein, albumin, globulin, aspartate transaminase and alanine transaminase. Yildirim–Aksoy, Eljack, Schrimsher, and Beck [22] also reported that hybrid tilapia (*O. niloticus* × *O. mozambique*) fed a 30% BSFLM diet for 12 weeks showed no influence on hematological values values. Likewise, Abdel–Tawwab, Khalil, Metwally, Shakweer, Khallaf and Abdel–Latif [7], found that no significant changes were observed in counts of WBCs, lymphocytes, monocytes, and neutrophils in BSFLM-fed fish as compared to the FM-fed fish. Conversely, Ushakova, Ponomarev, Bakaneva, Fedorovykh, Levina, Kotel’nikov, Kotel’nikova, Bastrakov, Kozlova, and Pavlov [41] observed increased hemoglobin in Mozambique tilapia (*O. mossambicus*) fed on a diet supplemented with dried black soldier flypre-pupae flour for one month.

The lysozyme activity can act as a non-specific molecule that beneficially protects the fish from the infectious disease through the breakdown of 1,4 glycosidic bonds present in the peptidoglycan of both Gram-positive and Gram-negative cell walls [73]. The enhancement of serum lysozyme activity will help in the stimulation of the fish’s immune responses and may contribute positively to the fish’s resistance against the challenged pathogens [24,74]. The results displayed an improved lysozyme and peroxidase activities in the skin mucus of fish fed 4% and 6% BSFLM, which refers to the enhanced immunity of fish in these groups. Xiao, Jin, Zheng, Cai, Yu, Yu, and Zhang [75] illustrated that the serum lysozyme activity of yellow catfish was not significantly different between the groups fed with BSFLM when compared with the control group. However, their values were increased over the control fish. In addition, Foysal, Fotedar, Tay, and Gupta [23] illustrated that marron (*Cherax cainii*) fed on both BSFLM supplemented diets showed significant enhancement of serum lysozyme activity. It has been reported that several fish species are able to synthesize endogenous chitinases, probably due to differences in their gut microbiota [51]. It is well known that dietary BSFLM has abundant amounts of chitin involved in increasing the abundance of microbial communities in the fish gut, thus acting as prebiotic substances that could induce immunostimulant impacts on fish [76,77]. However, further studies are required to find out the reasons for the enhanced immunity of fish fed BSFLM.

## 5. Conclusions

It can be concluded that BSFLM is regarded as one of the best alternatives for partial or complete replacement of FM in Nile tilapia diets. Fish fed the optimal level of included BSFLM can grow ideally without any adverse effects on the feed efficiency, somatic indices, and hematological parameters. The optimal level of included BSFLM also increased activities of lysozyme and peroxidase in the skin mucus. Based on the obtained results, BSFLM can completely replace FM in the diets of Nile tilapia without compromising the growth performance, feed efficiency, and health condition.

## Figures and Tables

**Table 1:** Ingredients and proximate composition of the experimental diets.

Ingredients (g/kg DM)	Diets						
	Diet 1	Diet 2	Diet 3	Diet 4	Diet 5	Diet 6	Diet 7
Fish meal (FM)	100	90	80	60	40	20	0
BSFLM ^1^	0	10	20	40	60	80	100
Corn meal	200	200	200	200	200	200	200
Soybean meal	450	440	435	415	400	380	365
Wheat flour	60	60	60	60	60	60	60
Rice bran	150	160	165	185	200	220	235
Cellulose	20	20	20	20	20	20	20
Soybean oil	5	5	5	5	5	5	5
Premix ^2^	10	10	10	10	10	10	10
Vitamin C ^3^	5	5	5	5	5	5	5
Proximate composition							
Dry matter (%)	97.15	97.05	96.95	96.95	97.10	96.85	96.95
Crude protein (%)	30.21	29.98	29.90	29.83	29.79	29.18	28.81
Crude fiber (%)	3.10	2.90	2.43	3.01	3.14	3.22	2.52
Crude lipid (%)	4.78	4.79	4.78	4.83	4.75	4.85	4.73
Ash (%)	6.92	6.62	6.17	6.16	5.65	5.39	4.95
Ca (%)	0.98	0.87	0.87	0.75	0.56	0.39	0.25
P (%)	1.00	1.03	0.97	1.00	0.90	0.92	0.93
Gross energy (kJ/g)	17.37	17.37	17.29	17.37	17.37	17.29	17.37

^1^ BSFLM = Black soldier fly larvae meal. ^2^ Vitamin and trace mineral mix supplemented as follows (IU kg^−1^ or g kg^−1^ diet): retinyl acetate 1,085,000 IU; cholecalciferol 217,000 IU; D, L-a-tocopherol acetate 0.5 g; thiamin nitrate 0.5 g; pyridoxine hydrochloride 0.5 g; niacin 3 g; folic 0.05 g; cyanocobalamin 10 g; Ca pantothenate 1 g kg^−1^; inositol 0.5 g; zinc 1 g; copper 0.25 g; manganese 1.32 g; iodine 0.05 g; sodium 7.85 g. ^3^ Vitamin C 98% 5 g.

**Table 2:** Composition of BSFLM.

Composition	Amount
Energy (Kcal)	461
Water (%)	23.33
Protein (%)	26.12
Fat (%)	36.47
Carbohydrate (%)	7.24
Dietary fiber (%)	3.62
Ash (%)	6.84
Lauric acid (%)	17.25
Total vitamin A (µg/g)	0
Vitamin B1 (mg/g)	0.26
Omega-3 (mg/g)	244.71
Omega-6 (mg/g)	2835.37
Omega-9 (mg/g)	4100.19

**Table 3:** Growth parameters (mean ± standard deviation, SD) after 12 weeks of feeding with control (0%BSFLM and 100% FM, and FM replaced at 10%, 20%, 40%, 60%, 80%, and 100% of the BSFLM.

Diets	IBW	FBW	DWG	WG	SGR	RGR	HPA	SR
1	14.62 ± 1.90	44.04 ± 3.52	0.35 ± 0.03	29.42 ± 2.19	1.32 ± 0.10	66.85 ± 2.67	0.88 ± 0.07	100
2	15.31 ± 2.24	45.25 ± 5.40	0.36 ± 0.04	29.95 ± 3.17	1.29 ± 0.03	66.25 ± 0.95	0.91 ± 0.11	100
3	14.72 ± 3.36	46.35 ± 0.71	0.38 ± 0.04	31.63 ± 3.02	1.39 ± 0.26	68.28 ± 7.02	0.93 ± 0.01	100
4	14.66 ± 2.82	47.52 ± 3.86	0.39 ± 0.02	32.86 ± 1.51	1.41 ± 0.15	69.32 ± 3.75	0.95 ± 0.08	100
5	14.91 ± 2.06	47.38 ± 4.22	0.39 ± 0.03	32.47 ± 2.87	1.38 ± 0.10	68.57 ± 2.66	0.95 ± 0.08	100
6	14.67 ± 2.61	44.58 ± 4.40	0.36 ± 0.04	29.91 ± 3.02	1.33 ± 0.16	67.16 ± 4.31	0.89 ± 0.09	100
7	14.51 ± 2.06	43.02 ± 3.15	0.34 ± 0.04	28.50 ± 3.37	1.30 ± 0.17	66.19 ± 4.70	0.86 ± 0.06	100
*p*-value	1.000	0.734	0.442	0.442	0.937	0.946	0.734	100
Means overall	14.77 ± 2.09	45.45 ± 3.61	0.37 ± 0.03	30.68 ± 2.82	1.35 ± 0.14	67.52 ± 3.64	0.91 ± 0.07	100

IBW (g) = Initial body weight; FBW (g) = Final body weight; DWG (g) = Daily weight gain (g per day); WG (g) = Weight gain; SGR (%/day) = Specific growth rate (% per day); RGR (%) = Relative growth rate; HPA = Harvest (kg per aquaria); SR (%) = Survival rate.

**Table 4:** Feed utilization (mean ± SD) after 12 weeks of feeding with control (0% BSFLM and 100% FM), and FM replaced at 10%, 20%, 40%, 60%, 80%, and 100% of the BSFLM.

Diet	Total Feed Intake (kg)	Rates of Feed Intake (g/fish/day)	Feed Conversion Ratio	Feed Efficiency (%)	Total Digestibility (%)	Apparent Protein Digestibility Coefficient (%)
1	1.96 ± 0.13	1.25 ± 0.08	2.22 ± 0.17	45.08 ± 3.32	47.65± 0.39	75.22 ± 0.52 ^d^
2	1.93 ± 0.07	1.24 ± 0.05	2.15 ± 0.27	46.92 ± 5.39	48.19± 0.37	76.20 ± 0.16 ^c^
3	1.99 ± 0.06	1.28 ± 0.04	2.15 ± 0.10	46.63 ± 2.03	47.83± 0.81	76.17 ± 0.30 ^c^
4	2.02 ± 0.12	1.29 ± 0.08	2.14 ± 0.31	47.38 ± 6.39	47.92± 0.00	77.45 ± 0.23 ^b^
5	2.03 ± 0.21	1.30 ± 0.13	2.16 ± 0.42	47.25 ± 8.31	48.72± 0.37	78.04 ± 0.27 ^b^
6	1.92 ± 0.06	1.23 ± 0.04	2.16 ± 0.17	46.53 ± 3.83	48.18± 1.14	77.38 ± 0.45 ^b^
7	1.91 ± 0.02	1.23 ± 0.01	2.23 ± 0.15	44.92 ± 2.90	48.19± 0.37	82.84 ± 0.60 ^a^
*p*-value	0.757	0.758	0.998	0.993	0.687	0.000
Means overall	1.96 ± 0.10	1.26 ± 0.07	2.17 ± 0.21	46.39 ± 4.31	48.09 ± 0.55	77.61 ± 2.41

a,b—Different superscript letters indicate statistically different values.

**Table 5:** Condition factor (CF), hepatosomatic index (HSI), and viscerosomatic index (VSI) after 12 weeks of feeding with control (0% BSFLM and 100% FM), and FM replaced at 10%, 20%, 40%, 60%, 80%, and 100% of the BSFLM.

Diet	Initial Total Length (cm)	Final Total Length (cm)	CF	HSI (%)	VSI (%)
1	9.73 ± 0.80	14.69 ± 0.75 ^b^	1.60 ± 1.60	1.67 ± 0.34	7.48 ± 1.52
2	9.84 ± 0.65.	15.30 ± 0.59 ^ab^	1.57 ± 0.19	1.63 ± 0.43	6.50 ± 1.29
3	9.40 ± 0.89	15.55 ± 0.72 ^a^	1.60 ± 0.06	1.68 ± 0.57	6.33 ± 1.15
4	9.71 ± 1.03	14.75 ± 0.74 ^b^	1.54 ± 0.08	1.39 ± 0.30	8.01 ± 2.28
5	9.51 ± 0.87	15.17 ± 0.88 ^ab^	1.60 ± 0.09	1.62 ± 0.38	6.10 ± 1.20
6	9.55 ± 0.78	14.69 ± 0.52 ^b^	1.61 ± 0.10	1.79 ± 0.50	7.23 ± 1.74
7	9.70 ± 0.60	14.91 ± 0.99 ^b^	1.62 ± 0.11	1.69 ± 0.48	6.67 ± 1.63
*p*-value	0.785	0.011	0.576	0.330	0.137
Means overall	9.63 ± 0.80	15.01 ± 0.80	1.59 ± 0.11	1.64 ± 0.44	6.90 ± 1.64

a,b—Different superscript letters indicate statistically different values.

**Table 6:** Blood parameters after 12 weeks of feeding with control (0% BSFLM and 100% FM), and FM replaced at 10%, 20%, 40%, 60%, 80%, and 100% of the BSF.

Diet	RBC	Hb	HCT	MCV	MCH	MCHC	RDW-CV	PLT
	10^6^/µL	g/dL	%	fl	pg	g/dL	%	10^3^/µL
1	1.46 ± 0.33	5.18 ± 1.37	23.24 ± 6.33	158.42 ± 19.69	35.36 ± 3.22	22.44 ± 1.53	12.41 ± 3.56	34.75 ± 43.05
2	0.77 ± 0.42	3.88 ± 2.42	12.90 ± 7.89	159.40 ± 22.48	48.82 ± 20.39	29.98 ± 9.14	10.45 ± 1.34	26.00 ± 4.18
3	1.29 ± 0.29	4.89 ± 1.29	20.17 ± 3.75	157.94 ± 18.11	38.23 ± 8.99	24.00 ± 3.10	11.68 ± 3.49	17.00 ± 12.73
4	1.23 ± 0.43	4.61 ± 1.57	20.03 ± 7.05	163.67 ± 21.82	37.95 ± 5.71	23.16 ± 0.75	14.30 ± 4.26	33.22 ± 37.68
5	1.01 ± 0.68	4.11 ± 1.92	16.10 ± 9.54	167.81 ± 19.46	48.93 ± 19.99	28.56 ± 8.95	16.97 ± 7.31	25.64 ± 25.49
6	1.24 ± 0.63	5.89 ± 2.70	22.40 ± 12.86	174.06 ± 23.98	48.01 ± 11.76	27.68 ± 6.04	15.01 ± 2.96	22.11 ± 11.50
7	1.07 ± 0.63	4.34 ± 2.58	17.03 ± 10.96	157.64 ± 20.84	43.13 ± 16.48	27.04 ± 8.23	11.17 ± 2.20	34.75 ± 34.34
*p*-value	0.340	0.547	0.378	0.639	0.236	0.171	1.69	0.85
Means overall	1.18 ± 0.52	4.78 ± 2.04	19.28 ± 9.09	163.11 ± 20.70	42.65 ± 13.52	25.92 ± 6.28	13.20 ± 3.88	27.97 ± 28.00

Red blood cell (RBC), hemoglobin (Hb), hematocrit (HCT), mean corpuscular volume (MCV), mean corpuscular hemoglobin (MCH), mean corpuscular hemoglobin concentration (MCHC), red blood cell distribution width (RDW-CV), Platelets (PLT). Also, no significant differences in white blood cell count (WBC), neutrophil, lymphocyte, and monocyte were detected between the control and BSFLM fed fish (Table 7).

**Table 7:** White blood cell count WBC differential after 12 weeks of feeding with control (0% BSFLM and 100% FM), and FM replaced at 10%, 20%, 40%, 60%, 80%, and 100% of the BSF.

Diet	WBC	Neutrophil	Lymphocyte	Monocyte
	10^3^/µL	%	%	%
1	3.59 ± 1.36	34.00 ± 26.25	50.78 ± 26.88	14.33 ± 15.51
2	3.05 ± 1.04	47.40 ± 24.94	36.80 ± 23.95	13.80 ± 5.45
3	3.58 ± 1.01	39.14 ± 19.34	47.00 ± 21.49	13.43 ± 9.52
4	3.87 ± 1.28	24.90 ± 13.64	63.03 ± 22.10	11.09 ± 12.49
5	4.25 ± 0.96	22.29 ± 18.95	63.00 ± 17.26	13.00 ± 8.87
6	3.59 ± 2.02	47.22 ± 33.32	40.33 ± 24.96	11.33 ± 10.91
7	3.69 ± 0.92	39.88 ± 21.57	45.25 ± 28.93	13.13 ± 10.43
*p*-value	0.838	0.261	0.260	0.996
Means overall	3.68 ± 1.28	35.95 ± 23.97	50.06 ± 24.59	12.77 ± 10.72

**Table 8:** Skin mucus lysozyme (SMLA) and skin mucus peroxidase (SMPA) after 12 weeks of feeding with control (0% BSFLM and 100% FM), and FM replaced at 10%, 20%, 40%, 60%, 80%, and 100% of the BSFLM.

Skin Parameters	Diet 1	Diet 2	Diet 3	Diet 4	Diet 5	Diet 6	Diet 7
SMLA	0.99 ± 0.04 ^c^	1.45 ± 0.05 ^b^	1.86 ± 0.07 ^b^	2.59 ± 0.12 ^a^	2.35 ± 0.09 ^a^	1.55 ± 0.21 ^b^	1.76 ± 0.13 ^b^
SMPA	0.09 ± 0.006 ^c^	0.12 ± 0.002 ^b^	0.13 ± 0.007 ^b^	0.18 ± 0.005 ^a^	0.17 ± 0.003 ^a^	0.12 ± 0.002 ^b^	0.11 ± 0.005 ^b^

a,b,c—Different superscript letters indicate statistically different values.

## Data Availability

The data presented in this study are available on request from the corresponding author.
